# 超高效液相色谱-线性离子阱/静电场轨道阱高分辨质谱法快速检测化妆品中22种功效成分

**DOI:** 10.3724/SP.J.1123.2022.03037

**Published:** 2022-09-08

**Authors:** Chensihui XIONG, Tianming DING, Jie LIU, Ou YI, Xiaoping DING, Yun XIE

**Affiliations:** 1.湖北中医药大学药学院, 湖北 武汉 430065; 1. School of Pharmaceutical Science, Hubei University of Chinese Medicine, Wuhan 430065, China; 2.湖北省药品监督检验研究院, 湖北 武汉 430075; 2. Hubei Institute for Drug Control, Wuhan 430075, China; 3.武汉美之修行信息科技有限公司, 湖北 武汉 437000; 3. Wuhan Beauty Evolution Information Technology Co., Ltd., Wuhan 437000, China

**Keywords:** 超高效液相色谱法, 线性离子阱/静电场轨道阱高分辨质谱法, 功效成分, 化妆品, ultra-high performance liquid chromatography (UHPLC), linear ion trap/orbitrap high resolution mass spectrometry (LTQ/Orbitrap MS), functional components, cosmetics

## Abstract

建立了超高效液相色谱-线性离子阱/静电场轨道阱高分辨质谱法同时测定化妆品中的22种功效成分。样品采用甲醇超声提取,C18色谱柱(100 mm×2. 1 mm, 1. 8 μm)分离,以0.1%(v/v)甲酸水溶液和乙腈为流动相进行梯度洗脱。在正离子模式下,以保留时间和一级母离子精确质量数进行定量分析,以高能碰撞诱导解离获得的二级碎片离子精确质量数进行确证。结果表明,该方法线性关系良好,检出限(LOD)为0.003~2.01 mg/kg;定量限(LOQ)为0.02~4.36 mg/kg;水、乳、霜3种基质中3个添加水平的回收率范围为63.2%~125.1%,相对标准偏差为0.18%~10.9%。对标示含有烟酰胺、抗坏血酸葡糖苷、咖啡因、泛醇及甘草类(光果甘草根茎叶、甘草根、胀果甘草根)、麦冬根、人参根、黄芪根、虎杖根、苦参根、地黄根、积雪草、茶叶提取物的54批样品进行检测,标示单体功效成分的样品均有检出,标示不同植物提取物的46批样品中,24批检出植物提取物的功效成分。该方法简便快速、定性定量可靠,适用于化妆品中22种功效成分的定量测定。

## Abstract (en)

Plant components from extracts of *Sophora flavescens*, rhodiola, ginseng, *Centella asiatica*, and tea play important roles in skin whitening, moisturizing, anti-aging, sun protection, anti-inflammation, antiseptic, bacteriostatic, and other effects of cosmetics. At present, no relevant standard methods have been established to detect the addition amounts of plant extracts in cosmetics. In addition, plant extracts listed in product labels may be undetectable due to their addition in trace quantities and the lack of technical support. Therefore, a quantitative method for the simultaneous determination of 22 functional components in cosmetics was established by ultra-high performance liquid chromatography-linear ion trap/orbitrap high resolution mass spectrometry (UHPLC-LTQ/Orbitrap MS). Target compounds were extracted with methanol from samples using ultrasonic extraction, and then separated on a C18 column (100 mm × 2.1 mm, 1.8 μm) with gradient elution of 0.1% (v/v) formic acid aqueous solution (A) and acetonitrile (B). The gradient elution program were as follows: 0-5 min, 5%B-8%B; 5-25 min, 8%B-60%B; 25-35 min, 60%B-80%B; 35-36 min, 80%B-5%B; 36-45 min, 5%B. The flow rate was 0.3 mL/min and the injection volume was 5 μL. Accurate masses of precursor ions were used to detect cosmetic functional components in positive ionization mode. The fragment ions obtained by higher energy collisional dissociation were used for confirmation of the functional components. Each compound showed good linearity. The limits of detection (LODs) were in the range of 0.003-2.01 mg/kg, and the limits of quantification (LOQs) were in the range of 0.02-4.36 mg/kg. Recoveries at three levels were 63.2%-125.1%, and relative standard deviations (RSDs) were 0.18%-10.9%. Fifty-four batches of samples labeled with four monomer functional components and nine plant extracts were tested. In the 17 batches of samples labeled with nicotinamide, 4 batches labeled with caffeine, and 6 batches labeled with *Sophora flavescens* root extract, the labeled functional components were detected. One out of 11 batches of samples labeled with D-panthenol was not detected. Three of the seven batches of samples labeled with ascorbyl glucoside were not detected. In the 21 batches of samples labeled with licorice extracts, the corresponding functional components were not detected in 9 batches. In the 21 batches of samples labeled with *Centella asiatica* extract, the corresponding functional components were not detected in 11 batches. In the 13 batches of samples labeled with tea extract, the corresponding functional components were not detected in 8 batches. In 11 of the 12 batches containing ginseng root extract, the corresponding functional components were not detected. In five of the six batches of astragalus membranaceus root extract samples, the corresponding functional components were not detected. In samples labeled with *Polygonum cuspidatum* root extract, *Rehmannia glutinosa* root extract, and *Ophiopogon japonicus* root extract, the corresponding functional components were detected. The method is simple, rapid, reliable, accurate, and suitable for the determination of the 22 functional components in cosmetics.

近年来,含有美白祛斑、抗衰单体成分和植物原料的化妆品受到了广大消费者的青睐,尤其是植物活性成分,因符合消费者天然的护肤理念,越来越多的商家将产品研发重心倾向于植物护肤和单体活性成分联合使用^[1]^。植物成分在皮肤美白、保湿、抗衰老、防晒、抗炎以及化妆品的防腐抑菌等方面发挥着重要作用,例如苦参、红景天、人参、积雪草、茶叶等提取物,具有美白祛斑、抗炎、抑菌、抗皱等作用^[2]^。此外,还有一些单体功效成分,在一定浓度范围内具有明显的保湿、美白祛斑、抗氧化作用,例如烟酰胺、抗坏血酸葡糖苷、咖啡因、泛醇^[3]^。化妆品因配方不同而植物提取物添加量各异,如何检测化妆品中植物提取物的添加量,进而与产品的相应功效关联,目前尚无相关标准方法。此外,因微量添加无法检测而存在的产品标签虚假标示因缺乏技术支撑而难以判定。

目前,化妆品中单体功效成分和植物活性成分的检测方法已有报道^[4⇓-6]^。HPLC方法已被用于单体功效成分烟酰胺、抗坏血酸葡糖苷、咖啡因等的检测^[7,8]^。LC-MS/MS方法被用于检测茶叶、甘草提取物等样品中的功效成分^[9,10]^。然而,LC-MS/MS适用于已知目标物的分析,在没有标准品和短时间内难以建立检测方法的情况下,很难快速完成检测。而兼具离子阱和高分辨质谱平行检测能力的线性离子阱/静电场轨道阱高分辨质谱检测系统(LTQ/Orbitrap MS),可以通过目标成分的一级精确质量数和二级碎片离子定性,无需对照品即可完成快速筛查和确证。

鉴于此,本研究运用超高效液相色谱(UHPLC)-LTQ/Orbitrap MS方法,采用全扫模式,建立了水、乳、霜基质类化妆品中22种功效成分的检测方法,为化妆品中功效成分的检测提供技术支持,更好地服务于化妆品监管工作。

## 1 实验部分

### 1.1 仪器与装置

Thermo U3000液相色谱仪,Thermo Scientific LTQ-Orbitrap XL组合式高分辨质谱仪,Thermo ST16台式离心机均购自美国Thermo Fisher公司;Vortex-Genic2旋涡混合器(美国Scientific Industries公司); Hyper Sonic DT-A超声波清洗仪(鼎泰恒胜公司); 45位N-EVAP氮吹仪(美国Organomation公司); Milli-Q A10超纯水机(美国Millipore公司); Mettler Toledo电子天平(梅特勒-托利多(上海)公司)。

### 1.2 主要材料与试剂

甲醇、乙腈(色谱纯,Merck公司);甲酸(色谱纯,阿拉丁公司)。

对照品:表没食子儿茶素(批号PF200906-11,纯度98.8%, CAS No. 970-74-1),甘草酸(批号Q5640050,纯度99.8%, CAS No. 1405-86-3),毛蕊花糖苷(批号F2190020,纯度98.4%, CAS No. 61276-17-3)购于上海安谱实验科技股份有限公司;表儿茶素没食子酸酯(批号PF201115-07,纯度98.74%, CAS No. 1257-08-5),咖啡因(批号SM201023-13,纯度100%, CAS No. 58-08-2),泛醇(批号SM190124-02,纯度98.30%, CAS No. 81-13-0),抗坏血酸葡糖苷(批号YT201008-15,纯度99.08%, CAS No. 129499-78-1),虎杖苷(批号PS210713-05,纯度99.87%, CAS No. 27208-80-6),黄芪甲苷(批号PF200906-11,纯度99.06%, CAS No. 84687-43-4)购于美国Stanford Chemicals公司;苦参碱(批号110805-202010,纯度98.7%, CAS No. 519-02-8),氧化苦参碱(批号110780-201909,纯度92.9%, CAS No. 16837-52-8),烟酰胺(批号100115-202005,纯度99.9%, CAS No. 98-92-0),短葶山麦冬皂苷C(批号111908-201102, CAS No. 130551-41-6),山麦冬皂苷B(批号111907-201804, CAS No. 87425-34-1),甘草次酸(批号110723-201715,纯度99.6%, CAS No. 471-53-4),甘草苷(批号111610-201908,纯度95.0%, CAS No. 551-15-5),大黄素(批号110756-201913,纯度96.0%, CAS No. 518-82-1),积雪草苷(批号110892-202006,纯度93.8%, CAS No. 16830-15-2),羟基积雪草苷(批号110893-202105, 纯度为93.7%, CAS No. 34540-22-2),人参皂苷Rb1(批号110704-202129,纯度94.3%, CAS No. 41753-43-9),人参皂苷Re(批号110754-202129,纯度96.0%, CAS No. 52286-59-6)购于中国食品药品检定研究院;麦冬皂苷D(批号G21O11L128330,纯度98.0%, CAS No. 41753-53-3)购于源叶生物科技有限公司。

### 1.3 标准溶液的配制

精密称取各对照品约5 mg用于配制标准溶液。抗坏血酸葡糖苷置于10 mL容量瓶中,用纯水溶解,再用甲醇定容;甘草酸置于另一10 mL容量瓶,用甲醇溶解后定容;两种标准溶液需临用新配。另将大黄素和泛醇置于同一10 mL容量瓶中,用甲醇溶解后定容;其他对照品置于同一10 mL容量瓶中,用甲醇溶解后定容;所有储备液保存于-20 ℃ 的冰箱中。

精密量取适量4种标准储备液,置于10 mL容量瓶中,用甲醇定容,得到质量浓度约为100 g/L的混合标准溶液,临用时,再用甲醇逐级稀释成所需质量浓度的混合标准溶液。

### 1.4 样品溶液的制备

准确称取样品约0.2 g置于5 mL容量瓶中,加入甲醇定容至5 mL,涡旋1 min,超声提取15 min,离心,取出上清液,部分样品需要在40 ℃氮吹后复溶到1 mL,再经0.22 μm微孔滤膜过滤到进样小瓶中,待测。

部分样品中待测物质含量因超出线性范围,采用甲醇稀释后进行测定。

### 1.5 仪器分析

#### 1.5.1 液相色谱条件

色谱柱:Xtimate UHPLC C18 (100 mm×2. 1 mm, 1. 8 μm,美国Welch公司);流动相:0.1%(v/v)甲酸水溶液(A)和乙腈(B);梯度洗脱程序:0~5 min, 5%B~8%B; 5~25 min, 8%B~60%B; 25~35 min, 60%B~80%B; 35~36 min, 80%B~5%B; 36~45 min, 5%B;流速:0. 3 mL/min;进样量:5 μL;柱温:30 ℃。

#### 1.5.2 质谱条件

电喷雾离子源(ESI)正离子模式;鞘气(N_2_)和辅助气(Ar)流速分别为40和10 arb (arbitrary unit);毛细管温度300 ℃;离子源电压2. 5 kV,电流100 μA;一级质谱质量扫描范围为*m/z* 100~1300,分辨率为60000;多级质谱采用高能碰撞诱导解离(HCD)模式,依赖一级质谱扫描中的第1到第4强峰,分辨率15000; HCD裂解能量为归一化能量35%。22种功效成分的质谱分析参数见表1。

**表 1:** 22种功效成分的UHPLC-LTQ/Orbitrap MS分析参数

Compound	Chemical formulae	t_R_/ min	M_r_	Theoretical [M+H/Na]^+^ (m/z)	Measured [M+H/Na]^+^ (m/z)	Product ions (m/z)
Nicotianamine (烟酰胺)	C_6_H_6_N_2_O	1.20	122.0475	123.0552	123.0560	80.0499, 96.0448
D-Panthenol (泛醇)	C_9_H_19_NO_4_	2.89	205.1309	206.1386	206.1401	76.0762, 188.1297
Caffeine (咖啡因)	C_8_H_10_N_4_O_2_	8.32	194.0798	195.0876	195.0887	138.0670, 180.0665
Ascorbyl glucoside (抗坏血酸葡糖苷)	C_12_H_18_O_11_	1.23	338.0844	339.0921	361.0766	199.0229, 185.0438
Glycyrrhetic acid (甘草次酸)	C_30_H_46_O_4_	28.33	470.3391	471.3468	471.3496	425.3441, 189.1653, 263.1662
Glycyrrhizinic acid (甘草酸)	C_42_H_62_O_16_	19.44	822.4032	823.4110	823.4744	453.3343, 734.4279
Liquiritin (甘草苷)	C_21_H_22_O_9_	12.66	418.1258	419.1336	419.1416	257.0817, 137.0241
Asiaticoside (积雪草苷)	C_48_H_78_O_19_	15.63	958.5131	981.5030	981.5079	493.1559
Madecassoside (羟基积雪草苷)	C_48_H_78_O_20_	14.83	974.5081	997.4979	997.5059	493.1553
Ginsenoside Re (人参皂苷Re)	C_48_H_82_O_18_	14.64	946.5495	969.5393	969.5474	789.4786
Ginsenoside Rb1 (人参皂苷Rb1)	C_54_H_92_O_23_	17.49	1108.6023	1131.5922	1131.5980	365.1078, 789.4811
Matrine (苦参碱)	C_15_H_24_N_2_O	1.79	248.1883	249.1961	249.1975	148.1132
Oxymatrine (氧化苦参碱)	C_15_H_24_N_2_O_2_	2.48	264.1832	265.1910	265.1926	247.1820, 205.1350, 148.1133
Epicatechin gallat (表儿茶素没食子酸酯)	C_22_H_18_O_10_	12.75	442.0894	443.0972	443.0912	139.0399, 123.0449, 153.0194
Epigallocatechin (表没食子儿茶素)	C_15_H_14_O_7_	7.63	306.0734	307.0812	307.0835	139.0398, 181.0508, 169.0508
Ophiopogonin D (麦冬皂苷D)	C_44_H_70_O_16_	23.57	854.4658	855.4736	855.4749	413.3059, 129.0555
Liriopesides B (山麦冬皂苷B)	C_39_H_62_O_12_	23.68	722.4235	723.4314	723.4337	269.1914, 415.2511
Liriope muscari baily saponins C	C_44_H_70_O_17_	22.53	870.4607	871.4685	871.4539	722.4424, 854.4653, 359.4652
(短葶山麦冬皂苷C)						
Polydatin (虎杖苷)	C_20_H_22_O_8_	12.60	390.1309	391.1387	391.1342	229.0835, 241.0878
Emodine (大黄素)	C_15_H_10_O_5_	23.40	270.0522	271.0601	271.0618	229.0521, 243.0672, 95.0135
Acteoside (毛蕊花糖苷)	C_29_H_36_O_15_	12.81	624.2048	647.1946	647.2004	501.1409
Astragaloside A (黄芪甲苷)	C_41_H_68_O_14_	18.41	784.4603	807.4501	807.4547	627.3914

## 2 结果与讨论

### 2.1 植物提取物和单体成分的选择

采用美丽修行大数据平台,对化妆品中使用频次较高的植物提取物和单体功效成分进行分析和统计(见图1),发现甘草类(光果甘草根茎叶、甘草根、胀果甘草根)、麦冬根、人参根、黄芪根、虎杖根、苦参根、地黄根、积雪草、茶叶提取物与烟酰胺、泛醇、咖啡因、抗坏血酸葡糖苷等单体成分在化妆品中使用较多,因此,本研究选择含有上述植物提取物和单体功效成分的产品为研究对象。

**图 1:**
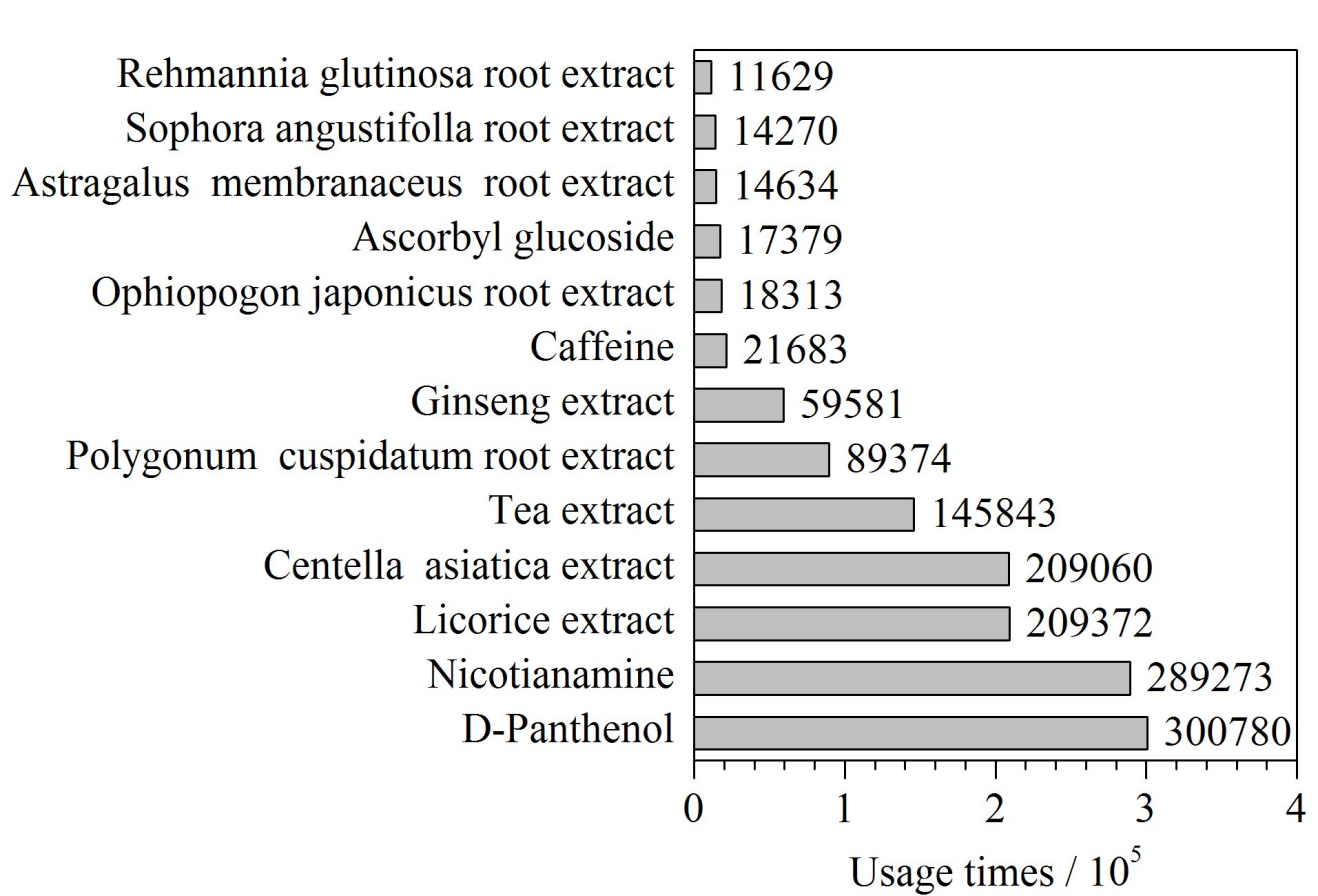
截至2021年12月美丽修行数据库中单体功效成分和 植物提取物在化妆品中的历史使用次数

鉴于植物提取物中成分复杂,根据文献^[11]^报道和2020年版《中国药典》中各药材的检测指标,积雪草提取物(*Centella asiatica* extract)的功效成分为积雪草苷和羟基积雪草苷;甘草类(光果甘草根茎叶、甘草根、胀果甘草根)提取物(licorice extract)的功效成分为甘草次酸、甘草酸、甘草苷^[12]^;麦冬提取物(*Ophiopogon japonicus* root extract)的功效成分为麦冬皂苷D、山麦冬皂苷B,短葶山麦冬皂苷C^[13]^;茶叶(茶)提取物(tea extract)的功效成分为咖啡因、表没食子儿茶素、表儿茶素没食子酸酯^[14]^;人参根提取物(ginseng extract)的功效成分为人参皂苷Re和人参皂苷Rb1^[15]^;膜荚黄芪根提取物(*Astragalus membranaceus* root extract)的功效成分为黄芪甲苷^[16]^;虎杖根提取物(*Polygonum cuspidatum* root extract)的功效成分为大黄素和虎杖苷^[17]^;苦参根提取物(*Sophora angustifolla* root extract)的功效成分为苦参碱和氧化苦参碱^[18]^;地黄根提取物(*Rehmannia glutinosa* root extract)的功效成分为毛蕊花糖苷^[19]^,选择上述植物中代表性成分对产品中添加的植物提取物进行筛查和检测。考虑到化妆品中植物提取物添加量低,选择植物提取物在外包装标签标示靠前的54批产品作为检测样品,其中水剂25批、乳剂19批,霜膏基质10批。

### 2.2 UHPLC-LTQ/Orbitrap MS分析条件的选择

首先考察了甲醇-0.1%甲酸水溶液、乙腈-0.1%甲酸水溶液两种流动相在相同梯度洗脱条件下,对物质分离的影响。结果表明,乙腈-0.1%甲酸水溶液作为流动相,能够较好地分离各物质成分,且峰形和分辨率较好。

在1.5.1节色谱条件下,优化干燥气温度、流速以及离子源温度等质谱参数。采用全扫模式可以获得各物质较好的峰形。在正、负离子模式下,采用归一化能量为30%、35%、40%和45%高能碰撞解离,获取二级碎片离子,结果表明在正离子模式下,归一化碰撞能量为35%时,多数化合物碎片离子丰富,便于确定目标成分。各物质的总离子流图及提取离子色谱图见图2。

**图 2:**
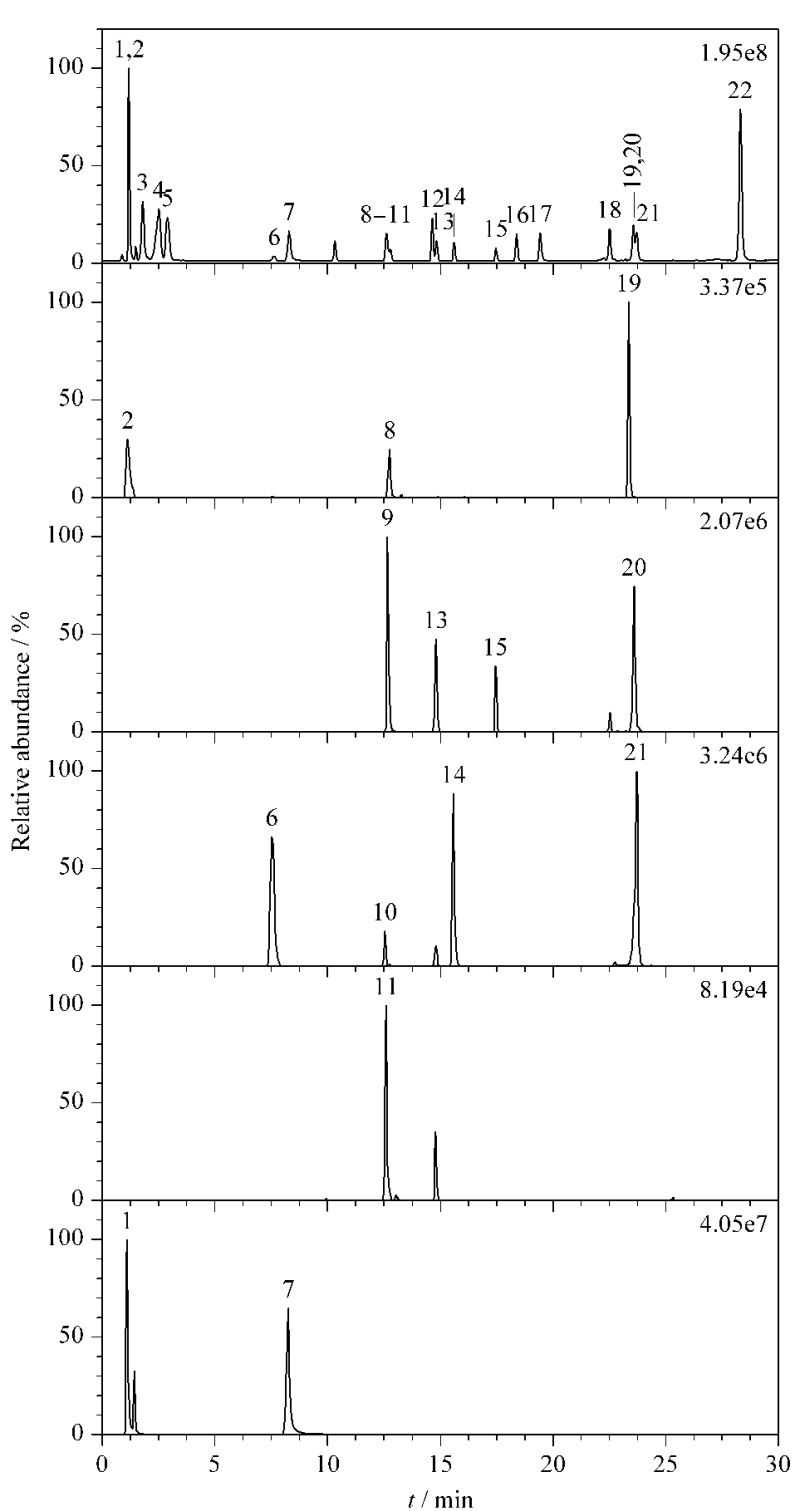
22种功效成分的总离子流图及提取离子色谱图

### 2.3 样品前处理条件的优化

以水、乳、霜为样品基质,分别选用不同体积分数的甲醇(20%、50%、80%、100%)水溶液进行提取,以加标回收率为指标考察提取效果。结果表明,采用甲醇-水体系时,当甲醇体积分数降低到50%时,提取液乳化现象严重,加入饱和氯化钠溶液且增加离心速率较难消除乳化效应,在乳、霜基质中100%甲醇的净化效果优于80%甲醇。根据功效成分的溶解性及回收率的结果,100%甲醇提取22种功效成分均能得到理想的提取效果。因此,本试验确定提取液为甲醇。

### 2.4 方法学评价

#### 2.4.1 线性关系和检出限

考察了混合标准溶液中各目标化合物的线性关系,以目标化合物的含量(*x*, mg/kg)和准分子离子峰面积(*y*)绘制标准曲线。各化合物的线性方程、相关系数(*R*^2^)、线性范围见表2。结果表明,各化合物的线性关系良好。由于高分辨质谱的基线噪声较低,以*S/N*方法计算出的检出限可能与方法的实际检测值存在差别,本研究采用混合标准溶液逐级稀释至仪器能检出的最低含量作为各化合物的检出限(见表2),定量限根据检出限经实测确定。

**表 2:** 22种成分的回归方程、相关系数、线性范围、检出限、定量限

Compound	Regression equation	R^2^	Linear range/(mg/kg)	LOD/(mg/kg)	LOQ/(mg/kg)
Nicotianamine	y=7.05×10^5^x+1.84×10^5^	0.9998	0.51-25.90	0.01	0.03
D-Panthenol	y=2.25×10^5^x+6.00×10^6^	0.9982	12.04-1204.43	0.03	0.07
Caffeine	y=5.91×10^5^x+1.35×10^4^	0.9997	0.05-25.00	0.01	0.03
Ascorbyl glucoside	y=1.34×10^4^x-3.11×10^4^	0.9994	2.48-123.85	1.25	2.34
Glycyrrhetic acid	y=8.93×10^5^x+2.23×10^5^	0.9999	0.05-12.65	0.003	0.02
Glycyrrhizinic acid	y=2.71×10^5^x-3.45×10^4^	0.9999	0.20-19.81	0.01	0.03
Liquiritin	y=5.13×10^2^x+5.49×10^3^	0.9994	11.16-223.25	1.12	3.36
Asiaticoside	y=2.79×10^4^x+5.73×10^3^	0.9992	0.41-10.38	0.04	0.12
Madecassoside	y=6.67×10^3^x+4.19×10^3^	0.9999	0.49-124.00	0.25	0.75
Ginsenoside Re	y=1.73×10^3^x+5.65×10^3^	0.9996	4.34-216.96	1.08	3.25
Ginsenoside Rb1	y=3.11×10^3^x+1.10×10^3^	0.9996	0.41-20.79	0.20	0.41
Matrine	y=6.12×10^5^x+8.00×10^6^	0.9991	12.14-242.80	0.01	0.03
Oxymatrine	y=6.97×10^5^x+1.19×10^5^	0.9997	0.04-21.09	0.01	0.02
Epicatechin gallat	y=2.42×10^4^x+2.04×10^3^	0.9993	0.25-25.03	0.05	0.15
Epigallocatechin	y=2.96×10^4^x-4.11×10^4^	0.9999	2.48-123.99	0.24	0.72
Ophiopogonin D	y=2.36×10^4^x+2.40×10^3^	0.9999	0.24-24.11	0.05	0.15
Liriopesides B	y=9.69×10^4^x+2.30×10^4^	0.9997	0.05-26.45	0.02	0.05
Liriope muscari baily saponins C	y=3.47×10^4^x-8.29×10^3^	0.9999	1.23-24.75	0.05	0.15
Polydatin	y=5.92×10^3^x+1.68×10^3^	0.9997	0.48-21.02	0.24	0.72
Emodine	y=7.92×10^3^x-1.03×10^4^	0.9992	4.02-40.17	2.01	4.02
Acteoside	y=3.49×10^3^x-4.64×10^3^	0.9989	4.36-43.64	1.23	4.36
Astragaloside A	y=1.19×10^4^x-6.50×10^2^	0.9999	1.33-53.29	0.13	0.32

*y*: peak area; *x*: content, mg/kg.

#### 2.4.2 方法回收率和精密度

精密称取乳剂、霜膏基质和水剂的空白样品0.2 g,分别添加低、中、高3个质量浓度水平的22种成分的混合标准溶液,根据建立的检测方法进行加标回收试验。每个质量浓度重复测定3次,计算回收率和相对标准偏差(RSD),水、乳、霜3种基质的加标回收率分别为63.2%~116.3%、63.6%~125.1%、68.9%~119.4%, RSD分别为0.18%~9.9%、0.78%~10.3%、0.78%~10.9%,结果表明,在3个不同水平下加标回收率均能满足实验要求。通过对乳剂加标回收率的结果分析,发现甘草苷、黄芪甲苷、短葶山麦冬皂苷C、麦冬皂苷D的回收率超过120%,对于这4种物质的测定采用了基质标准曲线对基质干扰问题进行校正,结果发现其加标回收率为84.6%~113.4%, RSD为1.1%~4.9%,表明在乳剂中几种成分存在一定的基质干扰。

选择线性范围内中浓度的混合标准溶液添加到水、乳、霜空白样品中,在相同条件下平行处理6份,以6次测定的峰面积计算方法精密度,水剂中22种成分的RSD为0.96%~6.4%,乳剂中22种成分的RSD为1.4%~6.7%,霜膏基质中22种成分的RSD为2.0%~10.5%。

### 2.5 样品测定结果

按照所建立方法测定了2.1节所述的54批样品,均未检出地黄根提取物、虎杖根提取物、麦冬根提取物的代表物质,另6种植物提取物和4种单体功效成分检测结果见表3。

**表 3:** 6种植物提取物和4种单体功效成分的检测结果

Labeled components and substances to be tested	Labeled samples /batch	Not detected /batch	Detected representative components /batch	Below the quantitative limit /batch	Content range/ (mg/kg)
Licorice extract	21	9			
Glycyrrhizinic acid			5	1	0.06-0.32
Glycyrrhetic acid			1	0	0.03
Liquiritin			0	0	
Glycyrrhizinic acid, glycyrrhetic acid			6	2 batches of glycyrrhetic acid	7.02-2116.10, 0.17-0.70
Centella asiatica extract	21	11			
Asiaticoside			3	0	0.25-437.32
Asiaticoside, madecassoside			7	2 batches of madecassoside	0.13-1195.08, 0.64-4221.37
Tea extract	13	8			
Caffeine			2	0	0.05-2.08
Epicatechin gallat, epigallocatechin			1	1 batch of epicatechin gallat	0.35
Caffeine, epigallocatechin			2	1 batch of epigallocatechin	0.40-1.04, 0.46
Ginseng extract	12	11			
Ginsenoside Rb1			1	1 batch of ginsenoside Rb1	
Ginsenoside Re			0	0	
Astragalus membranaceus root extract	6	5			
Astragaloside A			1	0	1.88
Sophora flavescens root extract	6	0			
Matrine			2	0	3.61-12.20
Oxymatrine			1	0	38.03
Matrine, oxymatrine			3	1 batch of oxymatrine	5.65-2794.00, 0.03-61.18
Nicotianamine	17	0	17	0	1019.06-41955.27
D-Panthenol	11	1	10	0	688.70-4518.41
Ascorbyl glucoside	7	3	4	0	0.82-2714.98
Caffeine	4	0	4	0	46.62-6003.74

54批样品检测结果发现,17批标示烟酰胺、4批标示咖啡因、6批标示苦参根提取物的样品均检出相应功效成分;11批标示泛醇的样品有1批未检出;7批标示抗坏血酸葡糖苷的样品有3批未检出;21批标示甘草类提取物(光果甘草根茎叶、甘草根、胀果甘草根)的样品有9批未检出相应功效成分;21批标示积雪草提取物的样品有11批未检出相应功效成分;13批标示茶(茶叶)提取物的样品有8批未检出相应功效成分;12批人参根提取物的样品有11批未检出相应功效成分;6批膜荚黄芪根提取物的样品有5批未检出相应功效成分。

综上所述,单体功效成分基本能够检出,而植物提取物的功效成分存在多批次未检出。由于目前植物提取物原料缺乏基于功效成分的检测方法和标准,导致无法对产品标示植物提取物进行质量控制,因此,建立基于功效物质的植物提取物原料及产品的检测方法及标准,为进一步规范市场行为、更好地服务化妆品监管,具有一定的意义。

## 3 结论

本研究建立了化妆品中22种功效成分的超高效液相色谱-线性离子阱/静电场轨道阱质谱检测方法,研究结果表明该方法灵敏、准确,重复性好,能够高效筛查和检测化妆品中22种功效成分,为化妆品中植物提取物和单体功效成分的检测以及化妆品监管工作提供了一种技术手段。
